# Variability of the impact of adverse events on physicians’ decision making

**DOI:** 10.1186/1472-6947-14-86

**Published:** 2014-09-25

**Authors:** Raluca Cozmuta, Peter A Merkel, Elizabeth Wahl, Liana Fraenkel

**Affiliations:** 1 Yale University School of Medicine, PO Box 208031, New Haven, CT 06520-8031, USA; 2 Division of Rheumatology, University of Pennsylvania, Philadelphia, PA 19104, USA; 3 University of San Francisco, San Francisco, USA; 4 Clinical Epidemiology Research Center, VA Connecticut Healthcare System, West Haven, CT 06516, USA

**Keywords:** Vasculitis, Decision-making, Drug toxicity, Cyclophosphamide, Rituximab, Best-worst scaling

## Abstract

**Background:**

Physicians frequently differ in their treatment recommendations. However, few studies have examined the reasons underlying these differences. The objective of this study was to examine whether physicians vary in the importance they attach to specific adverse events for two treatment options found in recent randomized controlled trials to have equivalent efficacy and overall toxicity.

**Methods:**

A Max-Diff survey was administered to physicians attending a national scientific conference to quantify the influence of 23 specific adverse events on decision making related to two treatment options for vasculitis. This approach was chosen because it results in greater item discrimination compared to rating scales. We used Hierarchical Bayes modeling to generate the relative importance score for each adverse event and examined the association between physicians’ characteristics and the five most influential factors.

**Results:**

118 physicians completed the survey. The mean age (SD) was 48 years (10); 68% were male and 81% reported spending the majority of time in clinical practice. There was significant variability in the ratings of the relative importance of all adverse events, except those that were mild and easily reversible. We found a positive correlation between increasing physician age with ratings of sepsis (r = 0.29, p = 0.002) and opportunistic infection (r = 0.23, p = 0.016), and an inverse association between age with progressive multifocal leukoencephalopathy (r = - 0.28, p = 0.003). Physician sex, work setting, location, and number of patients with vasculitis seen per year were not associated with the influence of specific adverse events on decision making.

**Conclusion:**

Our findings demonstrate that physicians differ substantially in how they perceive the importance of specific adverse events which may help explain observed unwarranted variability in physicians’ recommendations in clinical practice. Further efforts are needed to ensure that the reasons underlying variability in physicians’ recommendations are transparent.

## Background

Physicians often differ in their treatment recommendations. Extant reports of patients receiving significantly different recommendations by physicians practicing in different locations have been published both in the medical literature and the lay press. This variability persists even among experts who are well versed in the same literature as illustrated by the publication of conflicting guidelines by separate organizations [1]. However, few studies have systematically examined the reasons underlying these differences. Variability in recommendations likely occurs more often under conditions of uncertainty where there are few high quality studies available to inform decisions [2]. In these situations, indirect evidence is frequently relied upon to inform decision making in clinical practice. This is especially true for less common conditions, such as vasculitis.

Anti-neutrophil cytoplasmic antibodies (ANCA)-associated vasculitis (AAV) refers to a group of life- and organ-threatening diseases characterized by inflammation of small blood vessels, most commonly involving the skin, lungs, and kidneys. When vasculitis was first described, glucocorticoids were the only available treatment. The introduction of cyclophosphamide in the 1970’s resulted in significant improvements in overall survival of patients with vasculitis. However, cyclophosphamide is associated with significant toxicity including infection, infertility, and cancer, and physicians have long sought safer alternatives.

In 2010, two randomized controlled trials, Rituximab in ANCA-Associated Vasculitis (RAVE) and Rituximab versus Cyclophosphamide in ANCA-Associated Vasculitis (RITUXVAS) [3,4], demonstrated that rituximab is as effective as cyclophosphamide at inducing remission in patients with moderate to severe disease. Moreover, there were no differences in the rate of adverse events between the two groups. These results provided physicians with an alternative treatment option for AAV for the first time in approximately 40 years.

Given the equivalent rates of remission and toxicity induced by both treatment regimens, the decision of which treatment to initiate (assuming the patient has no contraindications to either option) is likely strongly influenced by judgments related to the impact of specific adverse events. The RAVE trial was designed as a non-inferiority trial because physicians believed rituximab to be a potentially safer alternative. This belief was based on a large body of indirect evidence generated from randomized trials and observational studies of patients with different diseases or using different treatment regimens demonstrating a lower risk of infection associated with rituximab compared to cyclophosphamide [5-26]. Moreover, unlike cyclophosphamide, rituximab is not associated with a risk of infertility or cancer. However, the RAVE and RITUXVAS trials, which are the only head-to-head comparisons of cyclophosphamide and rituximab in any disease, demonstrated no significant differences in toxicity between the two treatment arms, including serious infection or rates of hospitalization. Several of the major toxicities of concern for cyclophosphamide, including ovarian failure and bladder cancer, usually manifest after either higher cumulative doses of cyclophosphamide or longer follow-up times than present in the trials under discussion. Despite these limitations, the RAVE and RITUXVAS trials led not only to the approval of rituximab for use in AAV by the US Food and Drug Administration and regulatory agencies in other countries, but also rapid widespread adoption of the drug’s use as an remission induction agent in AAV.

**Figure 1:**
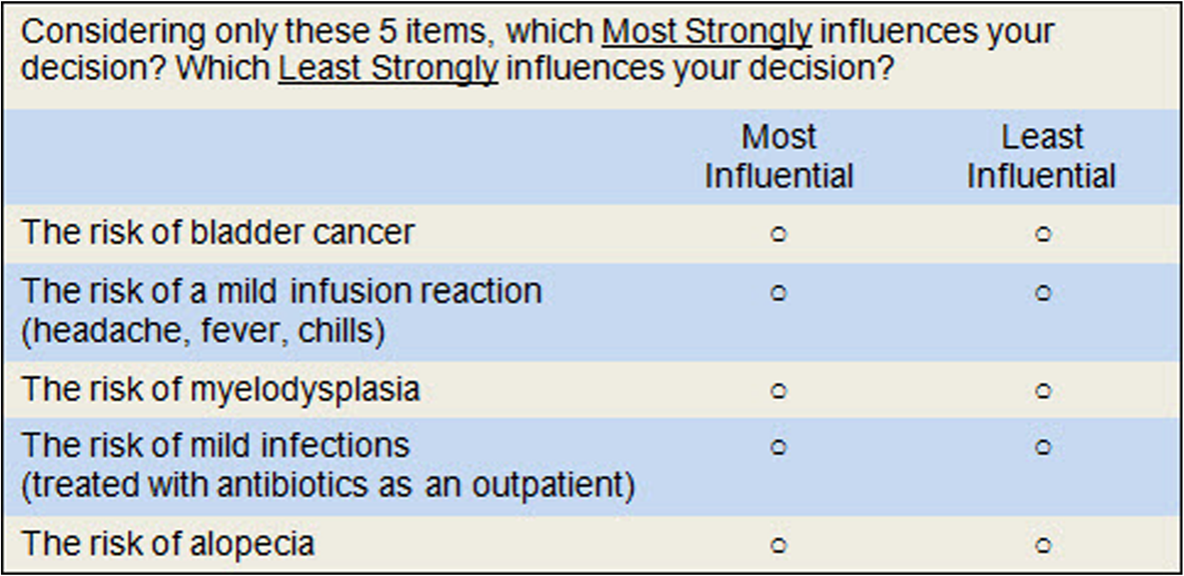
Example of choice task.

The objective of this study was to quantify the influence of specific adverse events on physicians’ treatment decisions for patients with newly-diagnosed AAV. Relative importance of specific attributes are most commonly measured using rating or ranking tasks. Visual analogue, Likert, or numeric rating scales are relatively easy to perform and permit inclusion of a large number of items. However, such rating scales are influenced by response biases, including social desirability bias and extreme response bias [27]. The latter is particularly problematic in healthcare research where subjects tend to rate all items as “Extremely Important”. Ranking tasks provide better discrimination, but significantly limit the number of items that can be included in a survey. In this study we used Sawtooth Software’s MaxDiff program as an alternative to a rating or ranking task. This approach asks respondents to choose the best (or the most important) item and/or the worst (or least important) item from a series of sets containing different combinations of items from a master list (see Figure 1 for an example). This method has several important advantages over rating scales including the ability to handle a large number of attributes, greater item discrimination, and avoidance of scale-related response bias [28].

## Methods

We administered a MaxDiff survey to a convenience sample of physicians attending the 2010 American College of Rheumatology National Scientific Meeting [29]. The survey was designed using Sawtooth Software (Sawtooth Software SSI Web version 6.4) and included 23 adverse events associated with either RTX and/or cyclophosphamide. Participants were given the following instructions:

“*On the following pages you will be asked to consider (given your knowledge about the severity and probability of each of the adverse events) how much each adverse event influences your decision about which medication to prescribe for a 50 year old post-menopausal woman with newly diagnosed severe ANCA-associated vasculitis. Assume the patient is hospitalized with pulmonary and renal involvement”.*We described the patient as being a postmenopausal woman so as to eliminate fertility concerns, which are extremely variable and difficult to control for in a standardized scenario. The survey asked respondents to choose the most important item from 20 sets containing different combinations of five items from the master list of 23 adverse events (see example in Figure 1).

The survey design ensured that each item 1) was shown approximately an equal number of times, 2) was paired with each other item an equal number of times, and 3) appeared in each position an equal number of times. We also collected data on the following physician characteristics: age, sex, number of years of experience, work setting, and the number of patients with vasculitis seen per year.

We used Hierarchical Bayes modeling to generate the mean relative importance score for each adverse event. The relative importance scores sum to 100, and reflect the impact of each characteristic on physicians’ decision making. For each respondent, utilities (zero-centered values) were calculated for each level of each attribute using Hierarchical Bayes (HB) modeling (Sawtooth CBC/HB system for hierarchical Bayes estimation version 4.0). HB modeling has the advantage that it can better incorporate heterogeneity between respondents’ choices. In HB modeling, the sample averages (prior information) are used to update the individual utilities in a number of iterations until the sample averages stop changing between iterations. After this convergence, the cycle is run several thousand more times and the estimates of each iteration are saved and averaged. Raw scores were rescaled to a 0 to 100 on a ratio scale [30]. The associations between physician characteristics and ratings were made using Pearson’s correlation coefficient and chi-square tests for continuous and categorical variables, respectively. This study qualified for exemption as determined by the Yale Human Investigation Committee.

## Results

One hundred and eighteen physicians completed the survey: 75 from North America and 22 from Europe. The mean age (SD) was 48 (10) years; 68% were male; 81% reported spending the majority of time in clinical practice; 39% work in an academic setting; 7% see fewer than 1 patient with AAV per year, 46% between 1 and 5 patients with AAV per year; 22% between 6 and 10 patients with AAV per year, and 25% see more than 10 patients with AAV per year.

**Figure 2:**
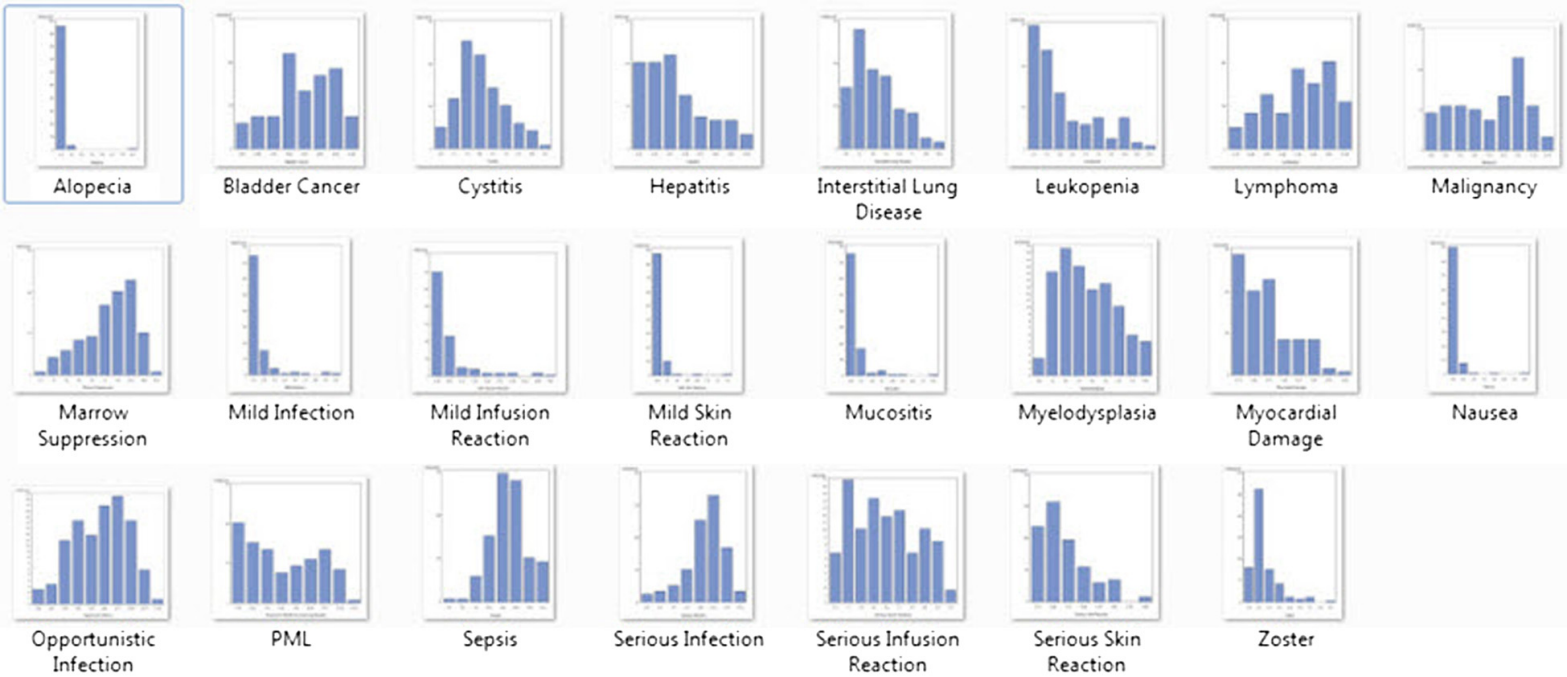
Snapshot of histograms representing variability in relative importance assigned to each adverse event.

**Figure 3:**
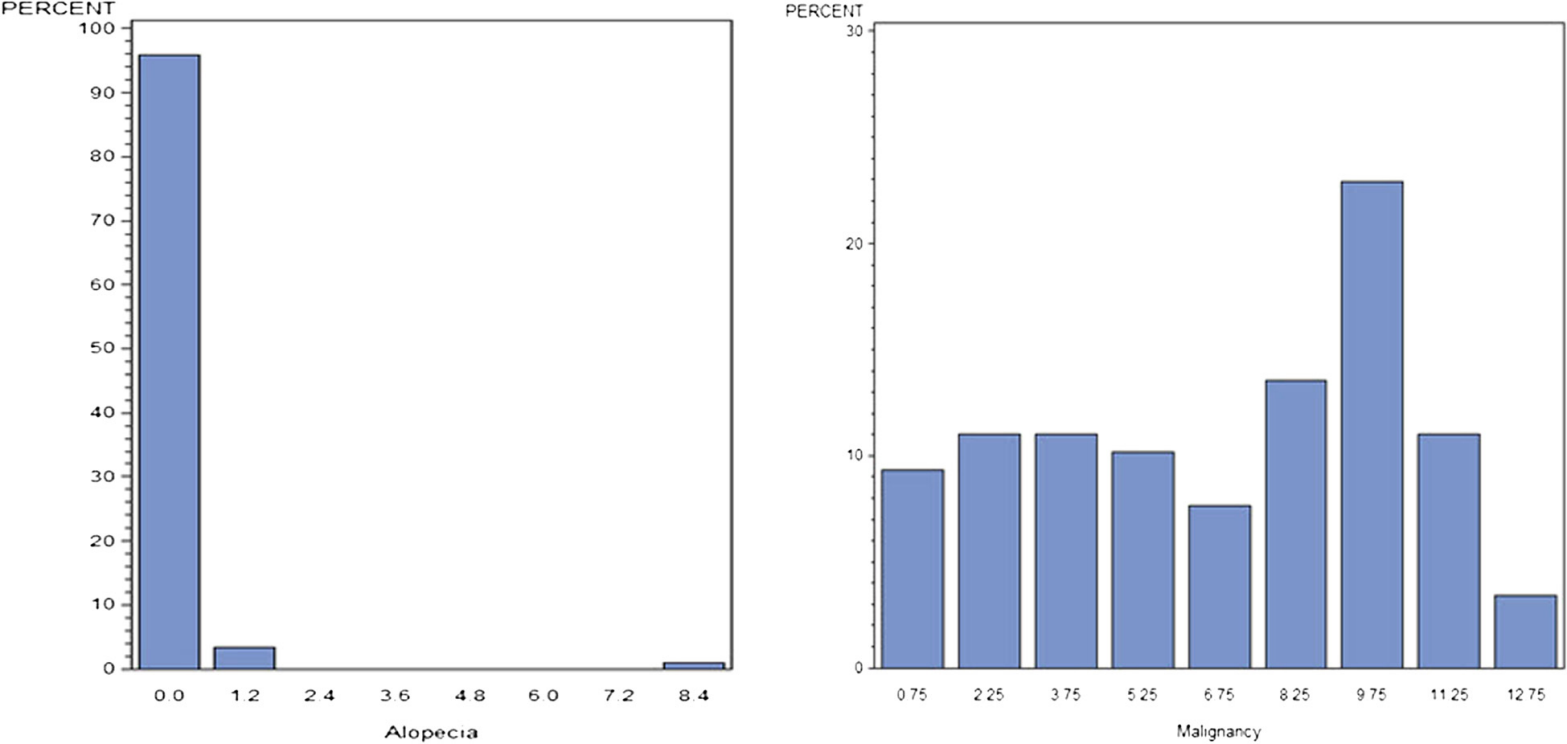
Relative importance assigned to a Mild AE (Alopecia) and a serious AE (Malignancy).

Physicians’ relative importance scores for each adverse event are provided in Table 1. Overall, physicians’ decisions were most strongly influenced by the risk of infection and cancer. As noted by the ranges in Table 1 and the histograms illustrating the distributions of the mean relative importance of each adverse event (Figure 2), there was significant variability in the ratings of all adverse events, except those that were felt to be the least important. Figure 3 provides a full sized example of a mild and serious adverse event.

**Table 1:** Relative importance scores for each adverse event

**Adverse events**	**Mean**	**Median**	**SD**	**Range**
Sepsis	11.3	11.3	1.6	5.4-14.7
Serious infection	11.0	11.3	2.1	4.0-14.8
Bone marrow suppression	8.0	8.5	2.9	0.3-13.8
Opportunistic infection	7.7	8.0	3.0	0.4-13.9
Lymphoma	6.9	7.2	3.1	0.1-11.3
Malignancy	6.7	7.7	3.5	0.1-13.1
Bladder cancer	6.6	7.0	2.8	0.1-11.5
Serious infusion reaction	5.8	5.7	3.8	0.1-13.7
Progressive multifocal leukoencephalopathy	5.3	4.6	3.7	0.03-13.4
Myelodysplasia	4.9	4.6	2.5	0.5-10.2
Hemorrhagic cystitis	4.6	4.3	2.6	0.2-12.2
Hepatitis	4.2	3.5	2.9	0.1-11.6
Myocardial damage	3.4	3.1	2.6	0.1-11.1
Interstitial lung disease	3.4	2.8	2.6	0.1-10.8
Leukopenia	3.4	1.8	3.5	0.03-13.9
Serious skin reaction	3.3	2.4	2.4	0.2-11.4
Zoster	1.8	1.2	1.7	0.05-10.0
Mild infection	0.5	0.1	1.1	0.004-6.7
Mucositis	0.4	0.1	0.8	0.002-6.7
Alopecia	0.2	0.03	0.4	0.0009-3.1
Nausea	0.1	0.03	0.8	0.002-8.4
Mild infusion reaction	0.06	0.02	0.1	0.001-0.5
Mild skin reaction	0.05	0.009	0.2	0.0008-1.5

**Figure 4:**
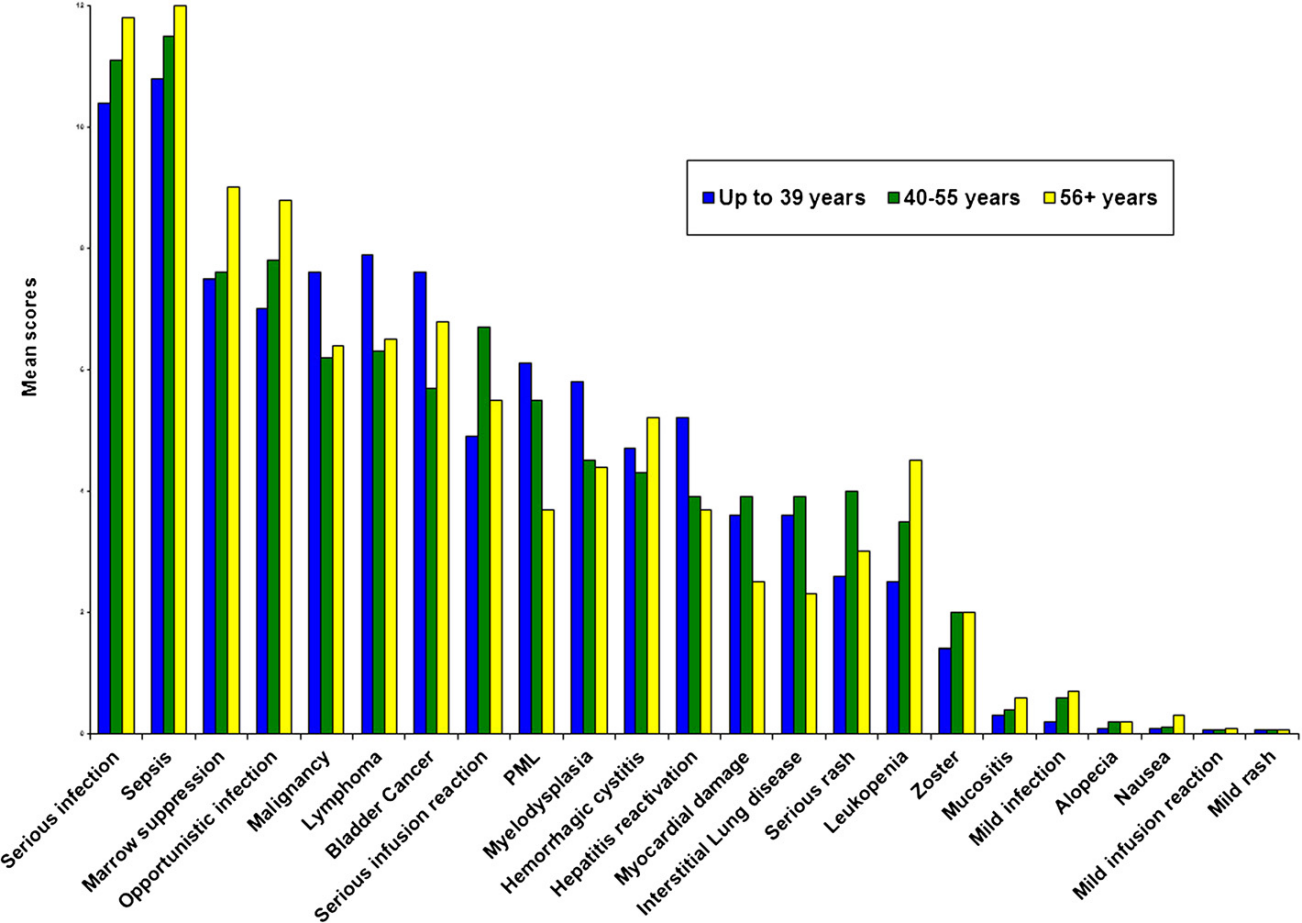
Mean relative importance scores by age group.

We examined the association between physicians’ characteristics and the five of the most influential factors: sepsis, lymphoma, opportunistic infection, serious infusion reaction, and progressive multifocal leukoencephalopathy. There was a positive correlation with increasing physician age with ratings of sepsis (r = 0.29, p = 0.002) and opportunistic infection (r = 0.23, p = 0.016). In contrast, older rheumatologists’ decisions were less influenced by progressive multifocal leukoencephalopathy (r = - 0.28, p = 0.003). The association between age and lymphoma was of borderline significance (r = - 0.19, p = 0.05) and age was not related to perceived importance of serious infusion reaction (r = 0.02, p = 0.9).Ratings of all adverse events by age group (up to 39, 40-55, 56+ years of age) are illustrated in Figure 4. Compared to younger physicians, older physicians were more strongly influenced by the risk of serious infection-related adverse events (except for progressive multifocal leukoencephalopathy) and less strongly influenced by malignancy. Physician sex, work setting, location, and number of patients with vasculitis seen per year were not associated with the impact of sepsis, lymphoma, opportunistic infection, serious infusion reaction, and progressive multifocal leukoencephalopathy on decision making.

## Discussion

The findings from this study demonstrate that physicians differ significantly in the impact that specific adverse events have on their decision making. With the exception of reversible and easily treatable adverse events, histograms showed wide distributions for all adverse events related to the two treatment options examined. Ratings were not related to clinical experience. However, we did find differences by age group, with physicians greater than 56 years old being more influenced by the risks of all infection-related adverse events, except for progressive multifocal leukoencephalopathy, than their younger counterparts. While the data do not explain the reasons underlying this pattern, it is possible that the differential impact between unspecified serious infections and progressive multifocal leukoencephalopathy is due to the relative recent attention focusing on the latter [31-33].

The RAVE [3] and RITUXVAS [4] head-to-head randomized controlled trials demonstrated no significant differences in the risk of infection between the two regimens. Despite these results, physicians ranked the risk of infection as the most important factor influencing their treatment decision. This finding was especially pronounced among older physicians. Physicians may undervalue risk estimates generated from randomized trials, because selective eligibility criteria, strict protocols, and short-term follow-up minimize adverse events. In addition, many of the risks associated with immunosuppressive medications are rare and would not be expected to occur in trials with relatively small numbers of patients. Small numbers and short duration of follow-up limit the usefulness of the toxicity data generated from head-to-head randomized controlled trials, however, the use of indirect data also has significant limitations. Most notably, the indirect data available for cyclophosphamide were generated from trials using higher doses of the drug and for much longer periods of time compared to the regimens currently used. This is particularly important because the most worrisome adverse events associated with cyclophosphamide (i.e. infection, infertility, and malignancy) are dose dependent.

There are several important limitations of this study. The use of standardized scenarios, enabled us to systematically vary patient characteristics, but this approach cannot replicate the complexity of decision making in clinical practice. In addition, although we surveyed a large number of physicians, most of whom spend the majority of their time in clinical practice, the study population represents a convenience sample of consecutively approached volunteers, which limits generalizability. Future research should examine whether differences in decision making predict differences in treatment choices made in clinical practice.

## Conclusions

Ideally, despite physicians differing in their judgments, patients should all receive the same information describing potential treatment options and the reasons underlying variability in physicians’ recommendation should be transparent. While we did not measure actual treatment decisions, these findings suggest that physicians differ substantially in how they perceive the importance of specific adverse events which may help explain the frequently observed unwarranted variability in physicians’ recommendations.

## Competing interests

Dr. Merkel has conducted research partially funded by Genentech, Inc.

## Authors’ contributions

RC participated in designing the survey, collecting and analyzing the data and writing the MS. PM participated in designing the survey, analyzing the data and writing the MS. EW participated in designing the survey, collecting the data and writing the MS. LF conceived of the study and participated in designing the survey, collecting and analyzing the data and writing the MS. All authors read and approved the final manuscript.

## Pre-publication history

The pre-publication history for this paper can be accessed here:

http://www.biomedcentral.com/1472-6947/14/86/prepub
